# The intra- and inter-rater reliability of five clinical muscle performance tests in patients with and without neck pain

**DOI:** 10.1186/1471-2474-14-339

**Published:** 2013-12-03

**Authors:** Tina Juul, Henning Langberg, Flemming Enoch, Karen Søgaard

**Affiliations:** 1 Institute of Sports Science and Clinical Biomechanics, The University of Southern Denmark, Odense, Denmark; 2 CopenRehab, Section of Social Medicine, Department of Public Health, Faculty of Health Sciences, University of Copenhagen, Copenhagen, Denmark; 3 FYSIQ Tårnby, Copenhagen, Denmark

**Keywords:** Physical therapy, Cervical spine, Assessment tools, Joint position sense

## Abstract

**Background:**

This study investigates the reliability of muscle performance tests using cost- and time-effective methods similar to those used in clinical practice. When conducting reliability studies, great effort goes into standardising test procedures to facilitate a stable outcome. Therefore, several test trials are often performed. However, when muscle performance tests are applied in the clinical setting, clinicians often only conduct a muscle performance test once as repeated testing may produce fatigue and pain, thus variation in test results. We aimed to investigate whether cervical muscle performance tests, which have shown promising psychometric properties, would remain reliable when examined under conditions similar to those of daily clinical practice.

**Methods:**

The intra-rater (between-day) and inter-rater (within-day) reliability was assessed for five cervical muscle performance tests in patients with (n = 33) and without neck pain (n = 30). The five tests were joint position error, the cranio-cervical flexion test, the neck flexor muscle endurance test performed in supine and in a 45°-upright position and a new neck extensor test.

**Results:**

Intra-rater reliability ranged from *moderate* to *almost perfect* agreement for joint position error (ICC ≥ 0.48-0.82), the cranio-cervical flexion test (ICC ≥ 0.69), the neck flexor muscle endurance test performed in supine (ICC ≥ 0.68) and in a 45°-upright position (ICC ≥ 0.41) with the exception of a new test (neck extensor test), which ranged from *slight* to *moderate* agreement (ICC = 0.14-0.41). Likewise, inter-rater reliability ranged from *moderate* to *almost perfect* agreement for joint position error (ICC ≥ 0.51-0.75), the cranio-cervical flexion test (ICC ≥ 0.85), the neck flexor muscle endurance test performed in supine (ICC ≥ 0.70) and in a 45°-upright position (ICC ≥ 0.56). However, only *slight* to *fair* agreement was found for the neck extensor test (ICC = 0.19-0.25).

**Conclusions:**

Intra- and inter-rater reliability ranged from *moderate* to *almost perfect* agreement with the exception of a new test (neck extensor test), which ranged from *slight* to *moderate* agreement. The significant variability observed suggests that tests like the neck extensor test and the neck flexor muscle endurance test performed in a 45°-upright position are too unstable to be used when evaluating neck muscle performance.

## Background

Neck pain is a common musculoskeletal complaint among adults. Worldwide estimates show that the 12-month prevalence of neck pain among adults ranges between 30% and 50%, depending on the definition of neck pain and the geographic spread of respondents [1]. At any given time, approximately 12-14% of the adult population reports having neck pain [1] and neck pain is now the second most common musculoskeletal disorder [2,3]. Likewise, neck pain often causes impairment, work disability and contributes to increased sickness absence [4,5] – thus millions of dollars are spent annually on treatment, compensation and lost earnings [6], and neck pain is a contributory cause of reduced health-related quality of life [7,8]. Neck pain has been associated with impaired performance of muscles in the cervical spine [9-13], as well as reduced proprioception and changes in the cervical motion patterns [14-17]. For this reason, treatment often includes exercise therapy aimed at restoring these neuromuscular deficits [18-23].

In order to assess any neuromuscular deficits present, it is of clinical importance to use reliable and valid assessment tools. Several performance tests have been developed with the aim of quantifying different aspects of muscle performance [24-33]. The present study focuses specifically on five muscle performance tests, which are often used in clinical practice.

The Cranio-Cervical Flexion Test (CCFT) is a clinical assessment test of the deep cervical flexor muscle function [28,30]. It targets activation and endurance of the deep cervical flexors in progressive inner range positions. The individual is placed in supine crook lying with the head in a neutral starting position, followed by an active head nodding action (cranio-cervical flexion) during which the patient tries to sequentially target five progressive stages (measured as an increased downward pressure of 22, 24, 26, 28 and 30 mmHg) [29,30]. The reliability of the CCFT has previously been assessed and it has shown promising psychometric properties [29,34-37]. Intraclass Correlation Coefficient (ICC) values have revealed *substantial* to *almost perfect* intra-rater reliability for the CCFT, with ICC values ranging from 0.78 to 0.98 (95% Confidence Interval (CI) ratings between 0.47-0.99) [24,29,35-37]. In addition, *moderate* to *almost perfect* inter-rater reliability has been reported, with ICC values from 0.57 to 0.91 (95% CI ratings between 0.37-0.96) [24,34,36].

Grimmer et al. [26] described a muscle performance test targeting neck flexor muscle endurance [26]. The test is performed with the subject in a supine crook lying position and measures the subject’s ability to maintain a cranio-cervical flexion (chin tuck), while performing an active head lift [26]. The maximal holding time is recorded in seconds. The recording is stopped when head movement, indicating fatigue occurs (i.e., inability to maintain upper cervical flexion, increase in neck flexion or lowering of the head). Reliability studies conducted on this muscle endurance test, as well as on several modified versions, have found *substantial* to *almost perfect* intra-rater reliability (ICC values from 0.71 to 0.96) [25-27,38-41]. Likewise, *moderate* to *almost perfect* inter-rater reliability has been reported (ICC values from 0.54 to 1.0) [27,39,40,42-44]. As patients with neck pain are often unable to perform the supine crook lying version, due to neck pain or reduced muscle strength, a modified version of the Neck Flexor Muscle Endurance (NFME) test is frequently used in clinical practice. The modified NFME test is performed in the same manner as the supine version [26,27] apart from the individual sitting in a 45°-upright position, which decreases the load on the neck. Nevertheless, little is known about the psychometric properties of the modified version.

Cervical Joint Position Error (JPE), measured as the ability to relocate the head to a starting position following active cervical range of motion, has been examined in patients with neck pain using several different measurement methods [16,32,33,45-48]. The test measures alterations in kinaesthetic awareness expressed as e.g. errors in head and neck repositioning. Studies using movement analysis devices, such as an ultrasound-based measuring device (Zebris) or electromagnetic tracking devices (3-Space Fastrak), have reported *substantial* to *almost perfect* intra- and inter-session reliability (ICC values from 0.61 to 0.84) [47,49-51], while others have failed to do so (ICC values from −0.01 to 0.51) [49,50,52,53]. Based on the results from e.g. Revel et al. [32] and Heikkilä et al. [45] it has been suggested that clinicians can use simple equipment such as a paper target and a head-mounted laser pointer to assess a subject’s ability to relocate the head to a neutral position following active cervical range of motion [54]. However, the reliability of such clinical performance tests is still unknown.

Over the last decade there has been an increased interest in muscle performance of the cervical flexors in patients with neck pain [12,21,30,55]. Muscle performance tests have focused predominantly on the cervical flexor muscles and only a limited number of tests targeting the posterior neck muscles exist [25,56]. However, recent research indicates that significant changes also occur in the posterior neck muscles [57-60], and there is a clinical need for the development of muscle performance tests targeting the posterior neck muscles. Drawing on the existing literature and the clinical practice we developed a new dynamic muscle performance test, which targets neck extensor muscle’ endurance.

When conducting reliability studies, great effort goes into standardising test procedures in order to reduce sources of variation and facilitate a stable outcome. One way to reduce test variation is by increasing the number of tests and using the average to calculate i.e. ICC values. Studies of muscle performance tests used for patients with neck pain have shown that an increased number of test trials (minimum of five trials) increases the test’s reliability (i.e., increased ICC values and decreased Limits Of Agreement (LOA)) [50,51] by reducing measurement error [61]. However, when muscle performance tests are applied in clinical practice, clinicians often only conduct a muscle performance test once or twice, partly due to time constrains and partly due to avoiding pain or fatigue in the tested muscles, which may affect test reliability (cf. increased measurement error).

Therefore, we aimed to investigate whether muscle performance tests, which have shown promising psychometric properties, remain reliable when examined under conditions similar to those of daily clinical practice in physiotherapy. Likewise, we aimed to target some of the areas where limited evidence exists. In order to standardise test procedures, we used inexpensive, simple equipment, which easily can be applied in a clinical setting and which previously has been found useful in tests of lumbar motor control [62].

The aim of this study was to determine the clinical reliability of five muscle performance tests in patients with and without neck pain.

## Methods

### Study design

An intra-rater (between-day) and inter-rater (within-day) design was applied. Each participant attended two assessment sessions. At each occasion both examiners assessed the participant. Intra-rater reliability on two days and was examined by comparing results from the two assessment sessions, with a maximum of three working days between the assessment sessions. Inter-rater reliability between examiner A and B was examined was assessed on both assessment sessions (first and second assessment session). The study followed a three-phase reliability protocol, recommended by the International Academy of Manual/Musculoskeletal Medicine (IAMMM) [63]. The three-phase protocol consisted of a preparation, training and an overall agreement phase. During the preparation phase agreements on study conditions and logistics were achieved, while the training phase focused mainly on replicating test procedures and judgment. The aim of the overall agreement phase was to obtain an overall agreement percentage >80% between the two examiners. After completing the three-phase protocol, both physiotherapists (examiners A and B) agreed upon how to determine a given cut-point (in case a clear cut off point did not already exist) and how to standardise and perform each test.

### Examiners

Between September 2011 and April 2012, two recently certified physiotherapists working at a private physiotherapy clinic (examiners A and B) examined 63 participants. A third physiotherapist (administrator) independently handled the administration of patients in terms of booking appointments and handing out questionnaires. The examiners were blinded to one another’s results and to whether the participant was a subject with or without neck pain. The order of examinations was random; that is, neither physiotherapist was consistently the first or the second examiner.

### Participants

The Regional Scientific Ethical Committee for Southern Denmark, approved the current study (reference number 30513). All participants gave written informed consent, and the rights of the participants were protected.

The participants consisted of two groups, who were either subjects with neck pain or a healthy reference group. Subjects with neck pain were recruited from five private physiotherapist clinics in Copenhagen, Denmark, and the physiotherapists’ consecutively referred patients, who fulfilled the inclusion and exclusion criteria. Healthy participants were recruited via advertisements in local newspapers or among friends or relatives of the three physiotherapists conducting the data collection. Patients with neck pain were eligible for participation if they met the following inclusion criteria: 1) had experienced non-specific neck pain for more than four weeks; 2) were over 18 years of age; 3) had turned to a general practitioner, chiropractor or physiotherapist regarding their neck pain; and 4) spoke and understood Danish. Patients were excluded if they had radiculopathy (e.g., positive Spurling’s Test, Upper Limb Tension Test [64,65]). Healthy subjects were eligible to participate if they: 1) were over 18 years of age; and 2) spoke and understood Danish. They were excluded if they: 1) had neck pain within the last year causing absence from work or a significant reduction in daily activity level for more than three days; 2) had back, shoulder or elbow pain; or had 3) a rheumatologic disease (e.g., rheumatoid arthritis). In addition, all participants were excluded if they had been diagnosed with a neurological disorder (e.g., Parkinson’s disease, multiple sclerosis), diabetes or cancer; 2) were pregnant; or 3) had a history of alcohol or drug abuse.

### Data collection

Participants were screened for eligibility before participating in the study. If the participants met the inclusion and exclusion criteria, arrangement for the first assessment was scheduled. The first assessment took place with a maximum of five working days between the screening session and the first assessment session. Referred patients received written information materials in hard copy at the clinics. Healthy participants received written information materials via e-mail. Prior to the first assessment session, study procedures were explained in detail to the participants, and participants gave their informed consent. The administrator collected information from participants regarding their gender, age and self-reported height, weight and education level. Neck pain was recorded using a 100 mm Visual Analogue Scale (VAS) anchored with “no pain” at 0 mm and “worst imaginable pain” at 100 mm. Participants completed the Neck Disability Index (NDI) [66], a questionnaire designed to measure Activities of Daily Living (ADL) in patients with neck pain. It consists of ten items, each with six response categories (range 0–5, total score between 0–50) [66].

After completing the questionnaire, participants performed the five clinical muscle performance tests with one examiner, followed by a short break (approx. 10 min.). After the ten-minute rest period, participants performed the same five clinical muscle performance tests with the second examiner. Each test session lasted approximately 30 minutes and the order of the five tests was random. Efforts were made to ensure that all subjects were examined at the same time of day at the first and second assessment session.

### Muscle performance tests

#### Joint position error (head repositioning)

The JPE test was a modified version of Heikkila and colleagues’ kinaesthetic sensibility test [45]. This test measures the subject’s ability to relocate their head to a starting position following active cervical range of motion in flexion, extension and bilateral rotation.

**Figure 1:**
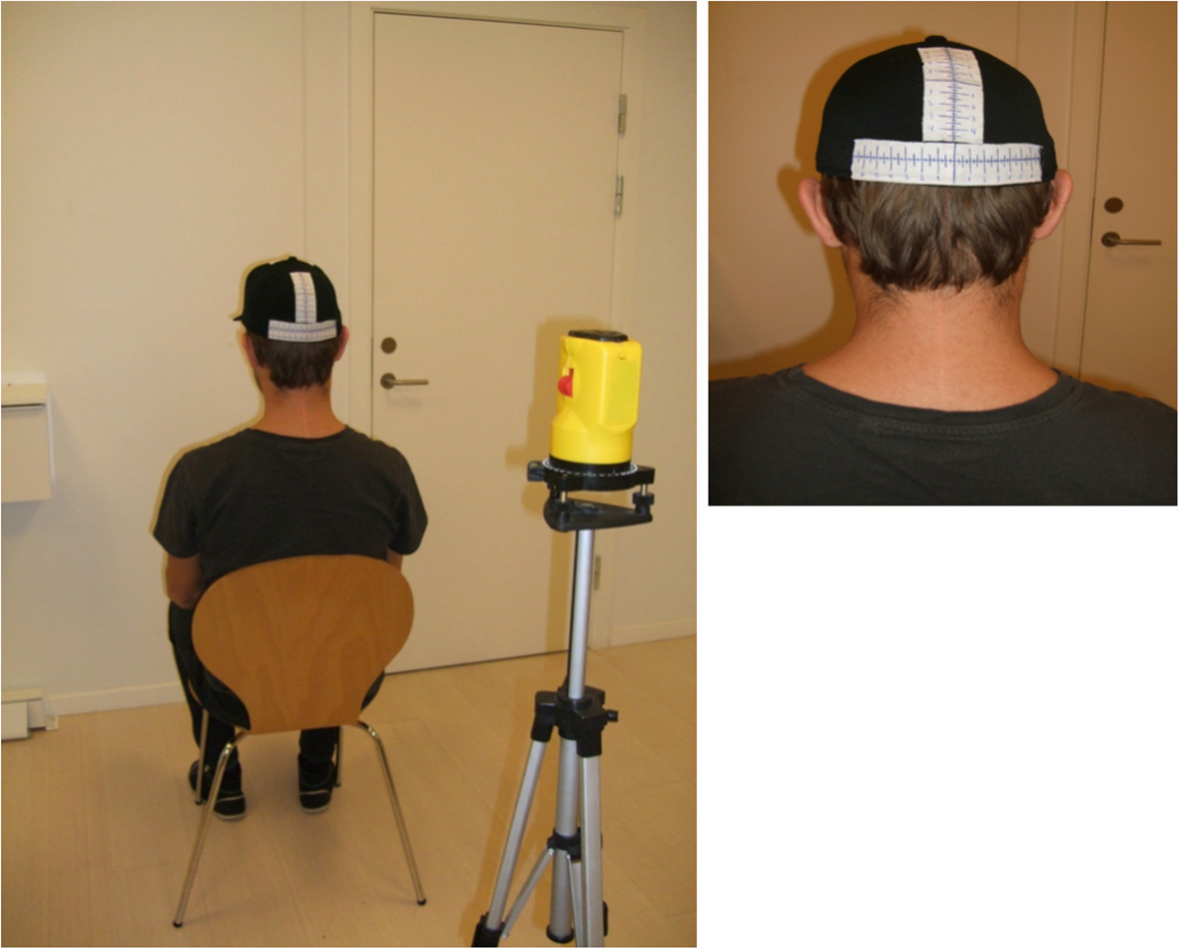
Start position for the joint position sense test.

In the modified JPE test, the subject wore headgear (a cap) with sagittal and a frontal measuring tape attached to the back (Figure 1). The tape had measurements at 0.25 cm intervals along a 12 cm length, starting with 0.0 cm in the middle and extending to 6 cm in both directions. The subjects were placed erect in a chair with back support and with approximately 90° of hip and knee flexion. The feet were firmly placed on the ground. A spirit level laser (Class 3A Laser product, Wen Zhou Xinke, China) was placed on a flat and stable surface behind the subject. The spirit level laser was positioned with the laser pointing at the centre of the measuring tape (i.e., at 0.0 cm). The starting position was sitting with the head in a neutral position (i.e., 0.0 cm) and with eyes closed. Subjects were asked to memorize this position. They maintained the position for a few seconds before performing a full active cervical rotation, followed by relocation of the head to the starting position. They were instructed to perform the test, as accurately as possible and to verbally indicate when they perceived having returned to their starting position. This position was recorded. The examiner registered the distance from the recorded position to 0.0 cm on the measuring tape. Between each trial, the examiner manually adjusted the participant’s head to match the original starting position (i.e., 0.0 cm) and gave no feedback on accuracy. No verbal or visual feedback was provided during the test. A familiarisation trial was conducted before the formal trial. The rate at which participants performed the movements was not formally controlled. However, all subjects were instructed to move at a comfortable pace. Participants performed a total of three trials of each movement direction in the following order: right cervical rotation; left cervical rotation; neck flexion; and neck extension.

#### Cranio-cervical flexion test

**Figure 2:**
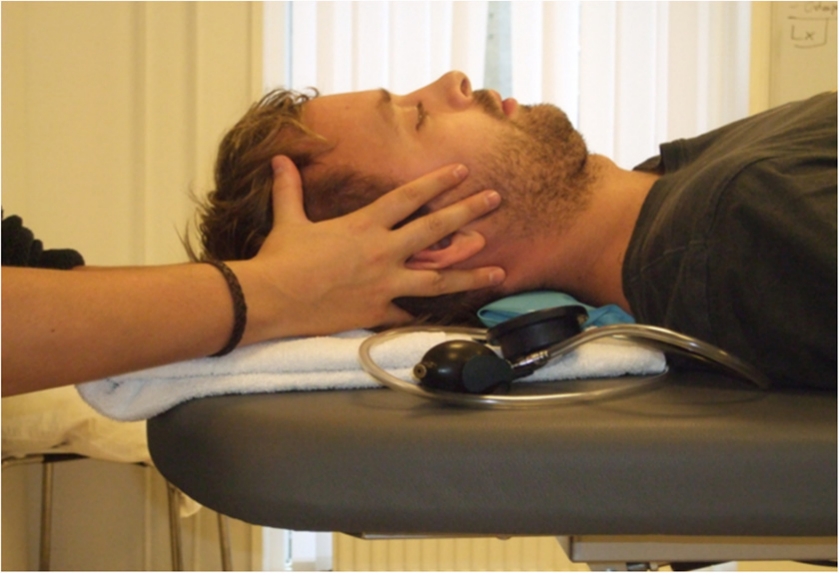
**The cranio**-**cervical flexion test.**

The CCFT is a clinical assessment of the deep cervical flexor muscles function [28,30]. The CCFT was performed with participants lying in supine crook on a plinth with the neck in a neutral position. Where necessary, head position was adjusted so the line of the face was horizontal by placing layers of towels under the head [30]. A deflated pressure biofeedback unit (Chattanooga Ltd Hixson, USA), with a pressure transducer attached, was placed underneath the neck abutting the occiput (Figure 2). It was inflated to a stable baseline pressure of 20 mmHg. Participants were instructed to perform a small, gentle and smooth nodding action (like saying ‘Yes’) to achieve cranio-cervical flexion. Progressive nodding action increased the pressure from the baseline of 20 mmHg to 22, 24, 26, 28 and 30 mmHg. Participants were instructed to maintain an isometric contraction at each progressed pressure level for ten seconds, before returning to a neutral position. A short break was given between each trail. Subjects were allowed one practice session to familiarise themselves with the test procedure and verbal feedback was provided to correct any incorrect movement strategies. The examiner observed the subject’s performance. When necessary, the examiner palpated the superficial neck muscles to ensure no use of incorrect movement strategies (e.g., undue use of superficial flexor muscles [e.g., m*.* Sternocleidomastoideus]*,* posterior retraction of the head, breath holding, overshooting of the target pressure). The examiner recorded which level of pressure the participant successfully achieve*.*

#### Muscle endurance tests

**Figure 3:**
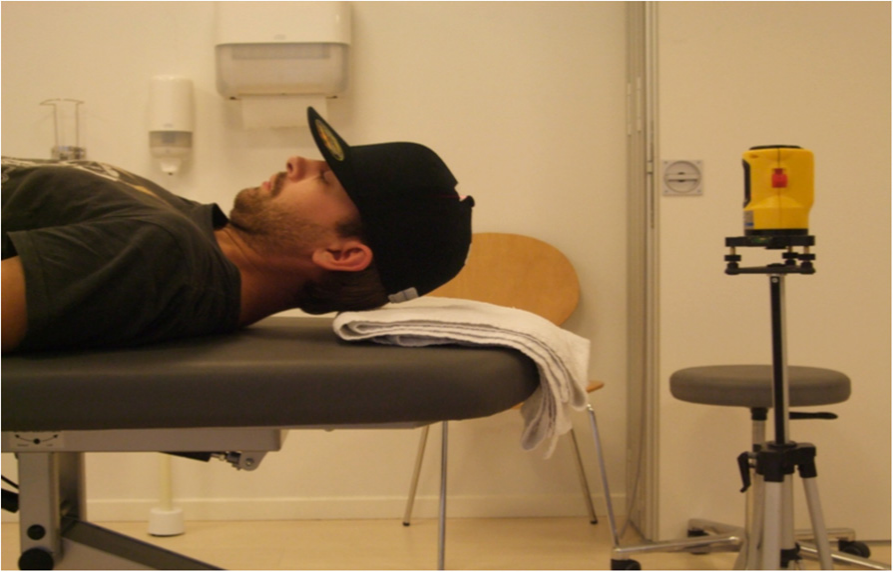
The neck muscle endurance test performed in supine.

The NFME test was based on a modified version of Harris et al. [27]. It is a clinical neck flexor muscle endurance test. The test was performed with the subject lying in supine crook on a plinth with the head in a neutral position (as during the CCFT). The participant wore headgear (a cap) with a 2 cm wide measuring tape applied to the top of the cap. A spirit level laser (Class 3A Laser product, Wen Zhou Xinke, China) was placed on a flat and stable surface above the subject (Figure 3). Initially, the participant was instructed to place their upper cervical spine in a slightly flexed position and gently lift their head off the plinth, while maintaining the upper cervical flexion. Subjects were allowed one short practice trial. The spirit level laser was positioned with the laser pointing at the centre of the measuring tape. The participant was instructed to hold the starting position for as long as possible. Verbal encouragement was given (e.g., “Hold your head up” or “Tuck your chin in”) if the participant started to change their head posture. The test was terminated when the laser moved outside either above or below - and thereby exceeded - the measuring tape due to head movement indicating fatigue (i.e., inability to maintain upper cervical flexion, increase in neck flexion or lowering of the head). The examiner recorded time to termination as the holding time in seconds. The participants performed this trial once.

**Figure 4:**
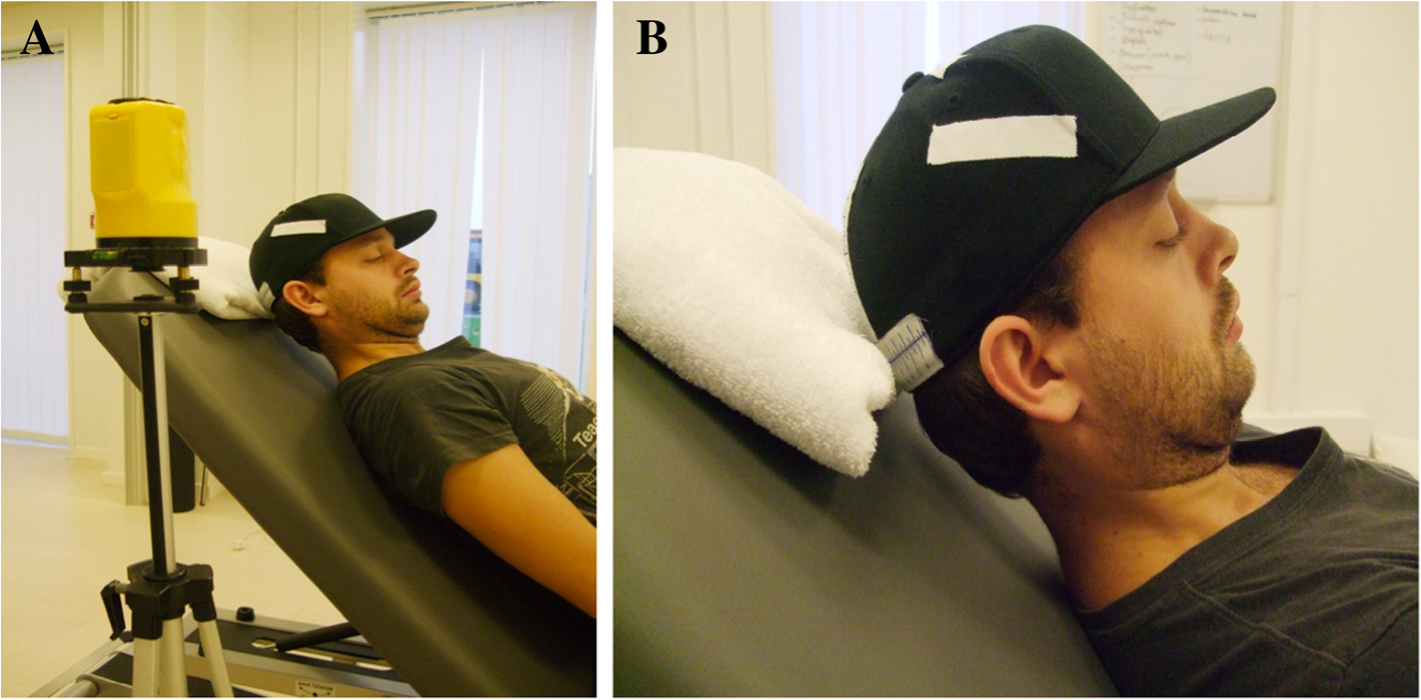
**The neck muscle endurance test performed in 45°-upright position** (**A) and (B).**

A modified NFME test was performed with the participant sitting in a 45°-upright position. The plinth served as back support (Figure 4A). The participant wore the same headgear, but with a 1.5 cm wide measuring tape applied on the side of the cap, approximately 2 cm above the right ear (Figure 4B). The spirit level laser was placed on the right side of the subject. The laser pointed at the centre of the measuring tape. Participants were allowed one short practice trial. Starting position was set as described above and the same instructions were given. The test was terminated when the laser moved outside either above or below - and thereby exceeded - the measuring tape due to head movement indicating fatigue (i.e., inability to maintain upper cervical flexion, increase in neck flexion or lowering of the head). The examiner recorded time to termination as the holding time in seconds. The participants performed this trial once.

#### Neck extensor test

**Figure 5:**
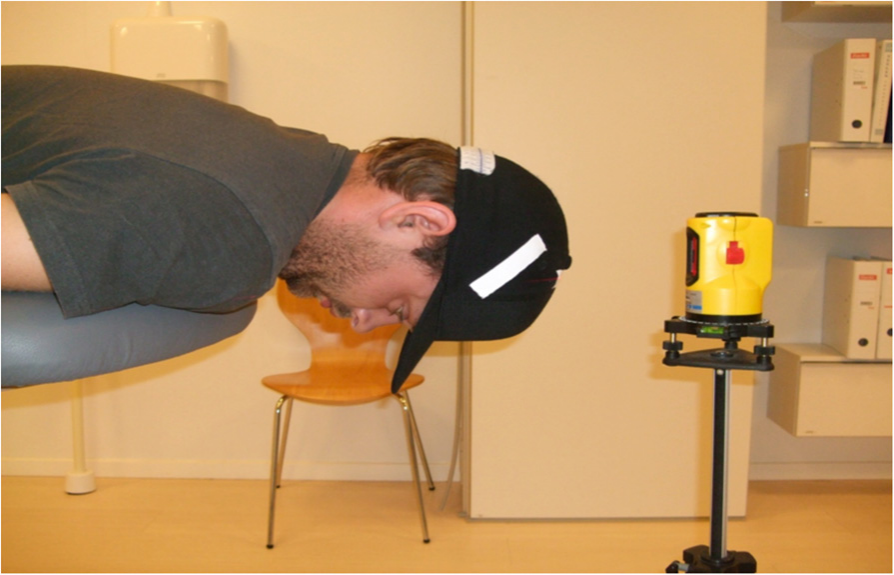
The neck extensor test.

The neck extensor test (NET) is a dynamic clinical test, which targets neck extensor muscle endurance*.* It was performed with the participant lying prone, with arms at the sides and the head over the edge of the plinth (Figure 5), initially supported by the examiner. The participant wore headgear (a cap) with a 2 cm wide measuring tape applied to the top of the cap. A spirit level laser was placed in front of the plinth (Class 3A Laser product, Wen Zhou Xinke, China). The examiner held the participant’s head in a neutral position, with the laser pointer at the centre of the measuring tape. The test began when the examiner stopped supporting the subject’s head. The participant was instructed to maintain a neutral head posture while performing a small side-to-side head rotation. They were told to perform the rotation at a smooth and slow pace. The rate at which participants performed the movements was not strictly controlled. However, all subjects were instructed to move at a comfortable pace. Participants were allowed one short practice trial. Verbal encouragement was given (e.g., “Hold your head up”), if the participant started to change their head posture. The test was terminated when the laser moved outside either above or below - and thereby exceeded - the measuring tape due to head movement indicating fatigue (i.e., inability to maintain upper cervical flexion, increase in neck flexion or lowering of the head). The examiner recorded time to termination as the holding time in seconds. The participants performed this trial once.

### Statistical analysis

Intra- and inter-rater reliability was assessed as recommended by the COnsensus-based Standards for the selection of health Measurement INstruments (COSMIN) checklist [61,67]. For assessing intra- and inter-rater reliability, ICC agreement values with 95% CI were calculated [61,67]. ICC agreement is preferred as it takes systematic and random errors into account [61]. Bland-Altman’s LOA [68] was used for evaluating agreement between the rater’s scores. Furthermore, measurement errors were estimated by calculating the Standard Error of Measurement (SEM) using formula: SEM consistency = SDdifference/√2 (SDdifference = Standard deviation of the mean differences between examiners A and B). The Smallest Detectable Change (SDC) was calculated using the formula: 1.96 * √2 * SEM [61,67].

Landis [69] criteria were used to interpret ICC agreement values: *slight* (r = 0.00-0.19); *fair* (r = 0.20-0.39); *moderate* (r = 0.40-0.59); *substantial* (r = 0.60-0.79); and *almost perfect* (r = 0.80-1.0) reliability [69]. Primary data analyses were performed for the whole group due to the small sample size. Data were analysed using SPSS version 19.0 (IBM®, SPSS, statistics). ICC agreement values (model 2.1.A) and 95% CI were calculated using ‘scale analysis’ with a two-way random effect model and ’absolute agreement’. For JPE, average measurements are reported. For the CCFT, the NFME and NET tests’ single measurements are reported. For head repositioning, no statistically significant differences were found between the three right and three left cervical rotation trials (post hoc analysis two-sample t-test, p = ≥0.70). Therefore, data from left and right cervical rotation were pooled in the final analysis. Adequate sample size is required to achieve an admissible 95% CI for ICC values and a sample size of 50 participants is recommended to assess reliability [70]. Additionally, a post hoc analysis was performed by a two-sample independent T-test to explore possible differences in mean scores between patients with neck pain and healthy subjects. This was done although the study was not designed with power to perform a strict specificity analysis. Statistical significance was accepted at P values less than 0.05.

## Results

A total of 63 subjects participated in the study. The descriptive characteristics of the 33 patients with neck pain and the 30 healthy subjects are provided in Table 1 with a summary of age, gender, height, body mass, body mass index, education level, VAS and NDI scores. Thirty healthy subjects (17 females/13 males) completed the first and second assessment sessions. Thirty-three patients with neck pain (25 females/8 males) completed the first assessment session and 31 patients with neck pain (23 females/8 males) completed the second assessment session. The two drop-outs were due to increased neck pain following the first assessment session and lack of time, respectively.

**Table 1:** Demographic characteristics of the patients with neck pain and healthy subjects*

	**Healthy subject** (**n** = **30**)	**Patients** (**n** = **33**)	**P**-**value for group difference**†
Gender, no. of females, %	17 ≈ 57%	25 ≈ 76%	0.108
Age, years	34 (14.7)	54 (15.1)	>**0.000**
Height, cm	173.7 (9.8)	171.9 (9.3)	0.468
Body mass, Kg	70.5 (12.8)	74.9 (15.9)	0.232
BMI, kg/m^2^	24.6 (3.3)	23.9 (3.3)	0.454
VAS pain score (0-100 score), mm	1.0 (3.5)	49.8 (21.5)	>**0.000**
NDI (0-50 score)	1.2 (2.8)	16.0 (8.1)	>**0.000**
Education level			
Ground school, number, %	2 ≈ 7%	10 ≈ 30%	
High school, number, %	14 ≈ 47%	12 ≈ 36%	
Undergraduate, number, %	10 ≈ 33%	7 ≈ 21%	
Graduate, number, %	4 ≈ 13%	3 ≈ 9%	
Doctoral degree, number, %	0	1 ≈ 3%	0.134

Cm = centimeters, Kg = kilogram, mm = millimeters, BMI = Body Mass Index, VAS = Visual Analogue Scale from 0-100 mm, where 0 represent the best result, i.e. more healthy. NDI = Neck Disability Index from 0-50, where 50 represents the best result, i.e. more healthy. Ground school = 9-10 years of education, High school = 10-12 years of education, Undergraduate = 13-15 years of education, Graduate = 15-17 years of education, Doctoral degree = 18-20% years of education. *Values are the mean ± SD unless otherwise indicated. † P values are based on 2-sample independent t-tests. Bold numbers = P values less than 0.05.

### Intra-rater reliability

Summarized statistics are presented for each of the muscle performance tests (examiners A and B) in Table 2. Overall, intra-rater reliability ranged from *slight* to *almost perfect* with ICC values between 0.14 and 0.82.

**Table 2:** **Intra**-**rater reliability of the five muscle performance tests**

**Examiner A**	**ICC agreement**	**95**% **CI**	**Meandiff**_**AB** (**SDdiff**_**AB**)	**SEM consistency**	**LOA**	**SDC**
JPE Rotation	0.66	0.43 - 0.79	0.08 (0, 28)	0.20	-0.468 0.634	0.55
JPE Flexion	0.82	0.71 - 0.89	-0.04 (0.31)	0.22	-0.640 - 0.560	0.60
JPE Extension	0.80	0.66 - 0.88	-0.004 (0.34)	0.24	-0.670 - 0.666	0.67
CCFT	0.69	0.53 - 0.80	-0.07 (2.61)	1.84	-5.176 - 5.044	5.11
Neck flexor muscle endurance (supine)	0.68	0.52 - 0.80	3.08 (23.13)	16.35	-42.25 - 48.41	45.34
Neck flexor muscle endurance (sitting 45°)	0.41	0.18 - 0.60	-18.21 (76.55)	54.13	-168.25 - 131.83	150.04
Neck extensor test	0.41	0.17 - 0.60	-0.07 (29.24)	20.68	-57.38 - 57.24	57.31
**Examiner B**						
JPE Rotation	0.70	0.50 - 0.81	0.05 (0.267)	0.19	-0.478 - 0.568	0.52
JPE Flexion	0.64	0.40 - 0.79	0.03 (0.344)	0.24	-641 - 0.717	0.67
JPE Extension	0.48	0.13 - 0.67	-0.03 (0.368)	0.26	-0.755 - 0.687	0.72
CCFT	0.81	0.70 - 0.88	0.00 (2.098)	1.48	-4.112 - 4.112	4.11
Neck flexor muscle endurance (supine)	0.75	0.61 - 0.85	-6.46 (20.61)	14.57	-46.86 - 33.94	40.40
Neck flexor muscle endurance (sitting 45°)	0.59	0.40 - 0.73	-10.97 (49.66)	35.12	-108.30 - 86.36	97.33
Neck extensor test	0.14	-0.12 - 0.37	-4.57 (32.58)	23.04	-68.43 - 59.29	63.86

JPE = Joint position error, CCFT = Cranio-Cervical Flexion Test. 95% CI = 95% confidence interval, ICC agreement = Inter class correlation coefficients. Meandiff_AB = mean difference between examiner A and B, SDdiff_AB = standard deviation of the mean difference between examiner A and B. SEM = standard error of mesurement, LOA = Limits of agreements, SDC = smallest detectable change.

#### Joint position error (head repositioning)

By and large, ICC values indicate *moderate* to *almost perfect* reliability for the JPE tests, ranking from 0.50 and 0.80. The highest ICC values were found for neck flexion (0.82 (95% CI [0.71-0.89]) and neck extension (0.80 (95% CI [0.66-0.88]) (examiner A), with 95% of the LOA measurement variation ranking between −0.640-0.666 cm (Table 2). However, examiner B presented the lowest ICC values for neck flexion (0.64 (95% CI [0.40-0.79]) and neck extension (0.48 (95% CI [0.13-0.67]). Bland-Altman plots revealed that the greater part of the differences between the two examiners was less than 1 cm for neck flexion and neck rotation. For neck rotation, ICC values implied *substantial* reliability for both examiners (Table 2). The SDC ranked from 0.52 cm (neck rotation) to 0.72 cm (neck extension) and SEM ranked between 0.19 cm (neck rotation) and 0.26 cm (neck extension) (Table 2).

#### Cranio-cervical flexion test

For the CCFT, the intra-rater reliability was *substantial* to *almost perfect*, with ICC values between 0.69 (95% CI [0.53-0.80]) and 0.81 (95% CI [0.70-0.88]). LOA ranked between −5.176-5.044 mmHg and −4.112-4.112 mmHg (Table 2), with a mean difference between examiners A and B of −0.07 mmHg (SD = 2.61) and 0.00 mmHg (SD = 2.10) (Table 2). Measurement errors expressed as SEM were 1.48 mmHg and 1.84 mmHg. The SDC was 4.11 mmHg and 5.11 mmHg.

#### Muscle endurance tests

Of the two NFME tests the supine version was the most reliable (Table 2). However, ICC values revealed only *substantial* intra-rater reliability (≤0.75 (95% CI [0.61-0.85]) (Table 2). The Bland-Altman analysis showed a very broad LOA, indicating limited agreement between the examiners (Table 2). Likewise, SEM disclosed large measurement errors (SEM ≥14.57 sec). The SDC on the NFME test (supine version) was above 40 sec (Table 2). When assessing the sitting version of the NFME test, intra-rater reliability was only *moderate* (≥0.41 (95% CI [0.18-0.60]) (Table 2). Similarly, mean differences (≥ −10.97 (49.66)) and LOA were large (≥ −108.30-86.36 sec) (Table 2). The SDC on the modified NFME test (sitting version) was above 97 sec (Table 2).

#### Neck extensor test

Overall, ICC values indicated *slight* to *moderate* intra-rater reliability for the NET. However, the 95% CIs were very large demonstrating significant variability (Table 2). Furthermore, broad LOA was observed, showing poor agreement between the variables (≥ −57.38- 57.24 sec) (Table 2). The SDC was between 57.31 sec and 63.86 sec.

### Inter-rater reliability

A summary of inter-rater reliability statistics is presented in Table 3. Generally, inter-rater reliability was *slight* to *substantial*, with ICC values ranking between 0.19 and 0.86 (Table 3).

**Table 3:** **Inter**-**rater reliability of the five muscle performance tests**

**First assessment**	**ICC agreement**	**95**% **CI**	**Meandiff**_**AB** (**SDdiff**_**AB**)	**SEM consistency**	**LOA**	**SDC**
JPE Rotation	0.51	0.19 - 0.70	0.04 (0.35)	0.50	-0.651 - 0.729	0.69
JPE Flexion	0.75	0.59 - 0.85	0.02 (0.31)	0.22	-0.560 - 0.628	0.61
JPE Extension	0.51	0.20 - 0.70	0.11 (0.38)	0.27	-0.644 - 0.862	0.75
CCFT	0.85	0.76 - 0.91	0.25 (2.31)	1.64	-4.281 - 4.789	4.54
Neck flexor muscle endurance (supine)	0.73	0.59 - 0.83	5.48 (21.56)	15.25	-36.78 - 47.74	42.26
Neck flexor muscle endurance (sitting 45°)	0.56	0.37 - 0.71	9.63 (51.32)	36.29	-90.96 - 110.22	100.59
Neck extensor test	0.19	-0.06 - 0.42	2.70 (30.94)	21.88	-57.94 - 63.34	60.64
**Second assessment**						
JPE Rotation	0.57	0.28 - 0.74	0.00 (0.28)	0.20	-0.544 - 0.546	0.55
JPE Flexion	0.71	0.52 - 0.82	0.06 (0.36)	0.25	-0.638 - 0.758	0.70
JPE Extension	0.69	0.48 - 0.81	0.10 (0.38)	0.27	-0.644 - 0.841	0.75
CCFT	0.86	0.81 - 0.93	0.33 (2.20)	1.55	-3.976 - 4.632	4.30
Neck flexor muscle endurance (supine)	0.70	0.55 - 0.81	-4.07 (22.51)	15.92	-48.19 - 40.05	44.12
Neck flexor muscle endurance (sitting 45°)	0.74	0.56 - 0.84	16.98 (49.71)	35.15	-80.45- 114.41	97.43
Neck extensor test	0.25	-0.01 - 0.47	-1.37 (33.35)	23.58	-66.74 - 64.0	65.37

JPE = Joint position error, CCFT = Cranio-Cervical Flexion Test. 95% CI = 95% confidence interval, ICC agreement = Inter class correlation coefficients. Meandiff_AB = mean difference between examiner A and B, SDdiff_AB = standard deviation of the mean difference between examiner A and B. SEM = standard error of mesuremeant, LOA = Limits of agreements, SDC = smallest detectable change.

#### Joint position error (head repositioning)

For the JPE tests, inter-rater reliability was moderate for neck rotation, with ICC values between 0.51 (95% CI [0.19-0.70]) and 0.57 (95% CI [0.28-0.74]), respectively (Table 3). Likewise, the ICC value for neck extension (first assessment) pointed to *moderate* reliability (0.51 (95% CI [0.20-0.70])). For the rest of the JPE tests, *substantial* reliability was observed (ICC ≥ 0.69) (Table 3), with SDCs between 0.55 cm and 0.75 cm. Overall, the mean differences between the two examiners ranked between 0.00 cm (SD = 0.28) and 0.11 cm. (SD = 0.38) (Table 3). Bland-Altman plots revealed that most of the differences for neck flexion and neck rotation were less than 1 cm.

#### Cranio-cervical flexion test

The ICC inter-rater reliability values were 0.85 (95% CI [0.76-0.91]) and 0.86 (95% CI [0.81-0.93]), indicating *almost perfect* reliability (Table 3). However, Bland-Altman analysis revealed a somewhat large LOA, signifying some inconsistency (Table 3). Likewise, SEM values were 1.55 mmHg and 1.64 mmHg, and the SDC was 4.30 mmHg and 4.53 mmHg, respectively (Table 3).

#### Muscle endurance tests

Apart from the second assessment of the sitting version of the NFME test, the overall inter-rater reliability for the NFME tests was *substantial* (ICC ≥ 0.70 (95% CI [0.55-0.81]) (Table 3). However, broad CIs were found, indicating variability. Similarly, the mean differences (from −4.07 to 16.98) and LOAs varied widely, from −36.79-47.74 sec to −80.45-114.41 sec (Table 3), indicating systematic errors between the two examiners. Significant measurement errors (expressed as SEM) were observed, especially for the sitting version of the NFME test (SEM ≥35.15 sec). The SDC ranged from 97.43 sec to 100.59 sec in the sitting version, and from 42.26 sec to 44.12 sec in the supine version (Table 3).

#### Neck extensor test

By and large, ICC inter-rater reliability values showed only *slight* to *fair* reliability for the NET (Table 3). LOA ranked between −57.94-63.34 sec and −66.74-64.0 sec (Table 3), with a mean difference between examiners A and B of −1.37 sec (SD = 33.35) and 2.70 sec (SD = 30.94), representing both systematic errors and large inconsistencies. The SEM showed considerable measurement errors (SEM ≥21.88 sec), with a SDC over 60 sec (Table 3).

#### Comparison of the results from the five muscle performance tests

Post hoc analysis was performed to compare mean scores between patients with neck pain and healthy subjects for each of the five muscle performance tests (Tables 4–5). For JPE, the only statistically significant differences found were in neck rotation and extension, where patients with neck pain showed significantly larger repositioning error than healthy subjects (p ≤ 0.023) (Tables 4–5). However, only examiner B found these significant differences and the differences observed for neck extension were only present at the second assessment session. Reduced neck flexor muscle endurance was shown in patients with neck pain, when compared with healthy subjects (p = 0.004). Nevertheless, reduced muscle endurance was only observed at the first assessment session (examiner A) and only when muscle endurance was measured in a 45°-upright sitting position.

**Table 4:** Differences in scores between patients with neck pain and healthy controls*

**Examiner A** - **First assessment**	**Control** (**n** = **30**)	**Patient** (**n** = **33**)	**P**-**value for group difference**†
JPE Rotation, cm	0.48 (0.30)	0.60 (0.31)	0.119
JPE Flexion, cm	0.50 (0.31)	0.66 (0.41)	0.095
JPE Extension, cm	0.56 (0.31)	0.70 (0.47)	0.164
CCFT, mmHg	26.93 (3.1)	24.97 (2.8)	**0.011**
Neck flexor muscle endurance (supine), sec	38.93 (28.9)	34.09 (37.1)	0.568
Neck flexor muscle endurance (sitting 45°), sec	103.40 (55.3)	63.18 (52.8)	**0.004**
Neck extensor test, sec	42.43 (24.8)	30.55 (24.4)	0.060
**Examiner B** - **First assessment**	**Control** (**n** = **30**)	**Patient** (**n** = **33**)	**P**-**value for group difference**†
JPE Rotation, cm	0.40 (0.21)	0.58 (0.36)	**0.016**
JPE Flexion, cm	0.54 (0.34)	0.59 (0.31)	0.615
JPE Extension, cm	0.51 (0.24)	0.55 (0.27)	0.515
CCFT, mmHg	27.07 (3.4)	24.36 (2.7)	**0.001**
Neck flexor muscle endurance (supine), sec	32.97 (18.8)	29.06 (31.2)	0.554
Neck flexor muscle endurance (sitting 45°), sec	84.67 (55.3)	61.82 (48.8)	0.087
Neck extensor test, sec	39.03 (29.3)	28.48 (15.6)	0.076

Cm = centimeters, mmHg = millimeter mercury, sec = seconds, JPE = Joint position error, CCFT = Cranio-Cervical Flexion Test. *Values are the mean ± SD. † P values are based on 2-sample independent t-tests. Bold numbers = P values less than 0.05.

**Table 5:** Differences in scores between patients with neck pain and healthy controls*

**Examiner A** - **First assessment**	**Control** (**n** = **30**)	**Patient** (**n** = **31**)	**P**-**value for group difference**†
JPE Rotation, cm	0.40 (0.17)	0.49 (0.31)	0.175
JPE Flexion, cm	0.52 (0.30)	0.66 (0.49)	0.186
JPE Extension, cm	0.58 (0.29)	0.71 (0.50)	0.225
CCFT, mmHg	27.67 (3.2)	24.32 (3.0)	>**0.000**
Neck flexor muscle endurance (supine), sec	37.20 (19.8)	30.97 (27.0)	0.309
Neck flexor muscle endurance (sitting 45°), sec	122.10 (74.6)	82.77 (86.0)	0.062
Neck extensor test, sec	40.77 (32.7)	33.39 (23.5)	0.314
**Examiner B** - **First assessment**	**Control** (**n** = **30**)	**Patient** (**n** = **31**)	**P**-**value for group difference**†
JPE Rotation, cm	0.36 (0.19)	0.53 (0.28)	**0.007**
JPE Flexion, cm	0.46 (0.30)	0.50 (0.38)	0.126
JPE Extension, cm	0.44 (0.20)	0.66 (0.46)	**0.023**
CCFT, mmHg	27.47 (2.6)	23.87 (3.1)	>**0.000**
Neck flexor muscle endurance (supine), sec	42.70 (21.8)	33.65 (42.5)	0.301
Neck flexor muscle endurance (sitting 45°), sec	95.60 (42.3)	75.00 (68.83)	0.166
Neck extensor test, sec	45.13 (29.4)	31.77 (19.9)	**0.042**

Cm = centimeters, mmHg = millimeters mercury, sec = seconds, JPE = Joint position error, CCFT = Cranio-Cervical Flexion Test. *Values are the mean ± SD. † P values are based on 2-sample independent t-tests. Bold numbers = P values less than 0.05.

For all CCFT measurements, patients with neck pain displayed significantly lower pressure levels than did healthy subjects (p ≤ 0.023), indicating a reduced ability to activate the deep neck flexors. For the rest of the measurements, no statistically significant differences were observed between patients with neck pain and healthy subjects.

In order to assess whether muscle fatigue introduced after performing the first set of muscle performance tests could have affected the reliability of the muscle endurance tests, a post hoc analysis was conducted comparing the mean holding time in seconds achieved from the first and the second assessment (on the same day). For the NFME test (supine), the NFME test (45°-upright position) and the NET, there were no statistically significant differences in holding time between the first and the second assessment on either of the two assessment sessions (p ≥ 0.190) (Table 6).

**Table 6:** Endurance measures from first and second assessment session*

	**First assessment**, **first test** (**n** = **63**)	**First assessment**, **second test** (**n** = **63**)	**P**-**value for group difference**†	**Second assessment**, **first test** (**n** = **61**)	**Second assessment**, **second test** (**n** = **61**)	**P**-**value for group difference**†
Neck flexor muscle endurance test (supine), holding time, sec	36.40 (33.25)	30.92 (25.92)	0.305	34.03 (23.74)	38.10 (33.94)	0.445
Neck flexor muscle endurance test (sitting 45°), holding time, sec	82.33 (57.23)	72.70 (52.83)	0.328	102.11 (82.34)	85.13 (57.81)	0.190
Neck extensor test, holding time, sec	36.21 (25.09)	33.51 (23.56)	0.535	37.02 (28.39)	38.34 (25.72)	0.787

Sec = seconds. * Values presented are mean ± SD. † P values are based on 2-sample independent t-tests.

## Discussion

This study was conducted in accordance with the COSMIN checklist and investigates the reliability of muscle performance tests using cost- and time-effective methods similar to those used in daily clinical practice in physiotherapy. Generally, across all tests the study showed large variability with intra- and inter-rater reliability ranging from *moderate* to *almost perfect* agreement with the exception of the NET, which ranged from *slight* to *moderate* agreement. In addressing why such significant variability was observed, several methodological issues and study limitations need to be considered.

### Joint position sense

Firstly, for head repositioning, the number of trials performed for each movement direction has been reported to affect the estimation of precision and accuracy, with an increasing test stability (i.e., higher ICC values) attained when a larger number of trials are performed (five trials or more) [50,51]. However, our results indicate that inter- and intra-rater reliability of neck rotation did not differ significantly from neck flexion or neck extension, despite the fact that calculations of ICC values for neck rotation were based on six trials (left and right), while ICC values for neck extension and neck flexion were only based on three trials each. A direct comparison to earlier studies should, however, be made with caution, since the methods of measurement are not directly comparable [50,51]. Secondly, age has been reported as one factor that can affect an individual’s ability to accurately reposition their head to a neutral position [71]. In the present study the patients are significantly older than the healthy subjects, which could have increased a difference in results. In spite of this the majority of our findings indicate that there are no significant differences between patients with neck pain and healthy subjects. Thirdly, a tendency to overshoot the target position has been found in patients with neck pain [32,45,71]. Unfortunately, data collection in the present study does not allow for investigation of a consistently over- or undershooting as part of the observed outcome variability. Fourthly, Treleaven et al. reported significantly larger errors in neck extension and rotation (to the right) in patients with whiplash when compared with controls [48]. However, our findings do not show a similar pattern. Only data from examiner B show significant differences between patients with neck pain and healthy subjects. Likewise, the differences observed for neck extension were only present at the second assessment session, not at the first assessment session. Possible explanations for these inconsistent findings include inadequate sample size and measurement error, since our study was not designed to detect differences between groups. Even though significant differences were found, the mean differences are all smaller than the tests’ measurement errors (Tables 2–3), which indicate that the differences observed may not be evidence of a true difference, but rather can be explained as measurement error. Therefore, our results should be interpreted with caution.

### The cranio-cervical flexion test

For the CCFT, our findings demonstrated *substantial* to *almost perfect* intra-rater reliability and *almost perfect* inter-rater reliability. These findings are consistent with the existing literature [29,34,36,37]. However, there is a tendency for higher ICC values to be reported with an increased number of trials performed [34,36,37]. When performing the CCFT, progressive nodding action increased the pressure from the baseline of 20 mmHg to 22, 24, 26, 28 and 30 mmHg. Despite the fact that the CCFT was found to be fairly reliable, the LOA and SDC were substantial (ranking between 4.11 and 5.11 mmHg). As a result, a change in score has to be at least 5 mmHg to be interpreted as a real change [61,72]. As previously reported [12,28,29,35], patients with neck pain demonstrated a reduced ability to activate the deep neck flexors, when compared with healthy subjects (Tables 4–5).

### Muscle endurance tests

The NFME test (supine version) has previously been found reliable [25-27,38-42]. Similarly, we found this test to have *substantial* inter- and intra-rater reliability. However, broad LOAs were determined for inter- and intra-rater reliability, indicating limited agreement between the examiners. SEM also revealed large measurement errors, with an estimation of 40 sec, estimated as the minimum detectable change. Edmondston et al. reported *almost perfect* intra-rater reliability with a minimum change of 17.8 sec representing a true change [25]. The mean holding time reported (≈50 sec) was almost twice the holding time reported in the current study (Table 6). However, their patient population was somewhat younger (mean age: 36 ±11) than the current patient population, which could explain the differences in holding time [73]. Previous studies have reported reduced holding time (i.e., reduced isometric neck flexor muscle endurance) in patients with neck pain, when compared with a healthy population (measured with the neck flexor muscle endurance test) [27,44]. All three muscle performance tests indicated a tendency towards shorter holding time in patients compared with healthy subjects, although the differences were not statistically significant (Tables 4–5). Due to the fact that patients with neck pain often are unable to perform the supine version of the NFME test, a modified version is often applied in clinical practice. The modified upright sitting version decreases the load on the neck, which for patients enables performance. By and large, our results imply that this modified version is not as reliable as the original supine version (Tables 2–3). The SDC for the sitting version was above 97 sec (Table 2), which is longer than the actual holding time observed for both healthy subjects and patients with neck pain, implying that changes in scores should be interpreted with caution. Possible confounding factors include the presence or increase of neck pain and the number of trials performed. Olson et al. [40] and Grimmer et al. [26] reported a systematic improvement in performance from a first to a second test [26,40] even through the tests were performed so close in time that no significant increase in muscle strength was expected. Such a learning curve could have affected the NFME test, increasing the variability of the test results. However, no statistically significant differences were found between the first and the second test indicating a learning curve did in fact not take place (Table 6).

### The neck extensor test

Despite the use of a standardised protocol, the overall level of reliability for the NET was poor, suggesting that this test is too unstable to be used to evaluate neck extensor muscle endurance. Several factors may have contributed to the discrepant findings. Firstly, some of the patients experienced increased pain during the muscle endurance performance tests and neck pain has in patients been shown to affect muscle performance [74,75]. Secondly, the order of the five muscle performance tests was random. Muscle fatigue has been found to influence muscle performance in patients with neck pain [76,77]. Theoretically, if the NET was performed last, muscle fatigue might have affected the outcome in both patients with neck pain and healthy subjects. However, post hoc analysis showed no statistically significant differences between the first and the second assessment performed on the same day (Table 6), which indicates that muscle fatigue did not influence the test results. Thirdly, even though great effort was invested into standardising the test protocol, it cannot be ruled out that discrepancy between test procedures could have affected the results.

### Test procedures

Test procedures for the CCFT, the NFME tests and the NET entailed each test only being performed once. This was done to replicate a clinical setting, where limited consultation time and the patient’s pain condition often confines the amount of test trials performed. In order to facilitate standardised test procedures that could be implemented in a clinic, we used inexpensive, easily accessible equipment, which allowed us, for example, to establish easily detectable cut off points at which muscle fatigue occurred and thereby reduce measurement error. Nevertheless, significant diversity was observed across the four muscle performance tests.

### Study strengths and limitations

The order of the examiner was random. This was done in order to avoid introducing measurement bias. However, some of the muscle performance tests aimed at measuring muscle endurance, which could have initiated muscle fatigue. If so, muscle fatigue would have occurred after performing the first set of muscle performance tests. This could theoretically have affected the outcome of the second set of muscle performance tests. Nevertheless, no statistically significant differences were found between the first and the second assessment for any of the muscle endurance tests (Table 6), which indicate that this was in fact not the case.

Despite a sufficient sample size (>50 participants) we found very broad 95% confidence intervals, which points to an inadequate sample size. A post hoc analysis was conducted to compare the results from patients with neck pain and healthy subjects. This was done in order to explore whether lack of variability among healthy subjects partly could explain our present findings. Furthermore, a difference between patients with neck pain and healthy subjects could point to relevant test candidates for future studies of specificity. However, due to the small sample size in the present study caution should be made when interpreting the results.

Inter-rater reliability reflects within-day comparison of the results. This may not mimic clinical practice as muscle endurance tests are often repeated after several days. Assessment of the between-day inter-rater reliability is likely to result in greater differences. Likewise, the use of recently certified physiotherapists may have contributed to the variation. More experienced clinicians might have achieved more reliable results, since the level of clinical skills needed to conduct the muscle performance tests are somewhat high. On the other hand recently certified physiotherapists may tend to follow the written protocol of procedures more strictly as they have no empirical routine to rely on. However, in both cases the findings presented in the present study are only related to test procedures performed in a similar manner. The present study replicated a clinical setting, with a broad range of therapists, including a large group with limited experience. An assessment tool has only limited clinical value if it takes years of practice to be able to reproduce stable results.

## Conclusions

This study investigates the reliability of five neck muscle performance tests using cost- and time-effective methods similar to those used in daily clinical practice in physiotherapy. Intra- and inter-rater reliability ranged from *moderate* to *almost perfect* agreement with the exception of a new test (neck extensor test), which ranged from *slight* to *moderate* agreement. The significant variability observed suggests that tests like the NET and the modified NFME test (sitting version) are too unstable to use when evaluating muscle performance. Furthermore, determining the smallest detectable change for the CCFT revealed that a change in score has to be at least 5 mmHg to be interpreted as a real change.

## Consent

Written informed consent was obtained from the patient for the publication of this report and any accompanying images.

## Abbreviations

COSMIN: COnsensus-based Standards for the selection of health status Measurement INstruments; ICC: Intraclass Correlation Coefficient; CI: Confidence Interval; SEM: Standard Error of Measurement; LOA: Limits Of Agreement; SDC: Smallest Detectable Change; CCFT: Cranio-Cervical Flexion Test; NFME: Neck Flexor Muscle Endurance (test); JPE: Joint Position Error; IAMMM: International Academy of Manual/Musculoskeletal Medicine; VAS: Visual Analogue Scale; NDI: Neck Disability Index; ADL: Activities of Daily Living; NET: Neck Extensor Test.

## Competing interests

The authors declare that they have no financial affiliation (including research funding) or involvement with any commercial organization that has a direct financial interest in any matter included in this manuscript. The authors declare that they have no conflict of interests.

## Authors’ contributions

TJ was involved in the planning of the study design, data acquisition, the data analysis and writing the paper. HL, FE and KS contributed to the analysis and interpretation of the data as well as study conception and design. All authors were involved in drafting the article or revising it critically for important intellectual content and all authors approved the final version of the manuscript.

## Pre-publication history

The pre-publication history for this paper can be accessed here:

http://www.biomedcentral.com/1471-2474/14/339/prepub
